# Automated measurements of greenhouse gases fluxes from tree stems and soils: magnitudes, patterns and drivers

**DOI:** 10.1038/s41598-019-39663-8

**Published:** 2019-03-08

**Authors:** Josep Barba, Rafael Poyatos, Rodrigo Vargas

**Affiliations:** 1 Department of Plant and Soil Sciences, University of Delaware, Newark, Delaware, 19716 USA; 2 CREAF, Cerdanyola del Vallès, 08193 Barcelona, Catalonia Spain; 3 Laboratory of Plant Ecology, Faculty of Bioscience Engineering, Ghent University, Ghent, 9000 Belgium

## Abstract

Tree stems exchange CO_2_, CH_4_ and N_2_O with the atmosphere but the magnitudes, patterns and drivers of these greenhouse gas (GHG) fluxes remain poorly understood. Our understanding mainly comes from static-manual measurements, which provide limited information on the temporal variability and magnitude of these fluxes. We measured hourly CO_2_, CH_4_ and N_2_O fluxes at two stem heights and adjacent soils within an upland temperate forest. We analyzed diurnal and seasonal variability of fluxes and biophysical drivers (i.e., temperature, soil moisture, sap flux). Tree stems were a net source of CO_2_ (3.80 ± 0.18 µmol m^−2^ s^−1^; mean ± 95% CI) and CH_4_ (0.37 ± 0.18 nmol m^−2^ s^−1^), but a sink for N_2_O (−0.016 ± 0.008 nmol m^−2^ s^−1^). Time series analysis showed diurnal temporal correlations between these gases with temperature or sap flux for certain days. CO_2_ and CH_4_ showed a clear seasonal pattern explained by temperature, soil water content and sap flux. Relationships between stem, soil fluxes and their drivers suggest that CH_4_ for stem emissions could be partially produced belowground. High-frequency measurements demonstrate that: a) tree stems exchange GHGs with the atmosphere at multiple time scales; and b) are needed to better estimate fluxes magnitudes and understand underlying mechanisms of GHG stem emissions.

## Introduction

Carbon dioxide (CO_2_), methane (CH_4_) and nitrous oxide (N_2_O) are the most important greenhouse gases, contributing 60, 20 and 10% to global warming, respectively^1^. Interactions between soil, vegetation and the atmosphere exert a crucial role controlling the global budget of these gases^2^. Particularly, forests influence GHG dynamics where their soils and leaves/canopies are active surfaces for GHG exchange^3,4^. Our current understanding of CH_4_ and N_2_O fluxes from forest ecosystems is mainly based on studies of forest soil measurements and canopies^2,5–7^. However, recent studies have revealed that stem surfaces could play an important role in regulating GHG fluxes^8–13^. For CO_2_, it is known that stem emissions are partially produced in the stem itself and partially produced in the rhizosphere and then, dissolved and transported upwards by stem sap flux^9^. However, much less is known about CH_4_ and N_2_O stem fluxes. CH_4_ in floodplain and wetland ecosystems is produced in soils under anoxic conditions and transported by roots to the stems^8,14,15^, but this direct relationship between soil and stem emissions is not as clear in upland forests. Several studies in the last 2 years have reported stem CH_4_ emissions in upland forests where adjacent soils are not a source but a sink of CH_4_^12,16–23^. Soils are often well aerated (specially in upland forests), and methanotrophic activity results in an uptake of 20 to 45 Tg CH_4_ y^−1^ at the global scale^24,25^. For N_2_O, the link between stem and soil dynamics is even less clear. Globally, soils are N_2_O sources (6.6 Tg N_2_O y^−1^)^26^ but in some cases they can act as sinks^27–29^. In contrast, stems have been described both as N_2_O sinks or sources, but this information is limited to very few studies^20,22,30–32^. Arguably, there are three key issues related to CH_4_ and N_2_O stem emissions in upland forests that represent a forefront of research^33^.

First, the magnitudes and patterns of stem emissions of CH_4_ and N_2_O in upland forests are poorly known. The few available studies suggest that there is a large variability of emissions between stems within mixed stands^12,17^, but also a large variability between trees from the same species^18,20,21,32^. Furthermore, these studies have not identified the main environmental drivers (e.g., temperature, soil moisture/precipitation) and the temporal dynamics of these GHG emissions.

A second issue deals with the origin (i.e., production and transport) of stem CH_4_ and N_2_O emissions in upland forests. Are these gases largely produced in the soil and transported upwards through the stem or are they mainly locally produced within the heartwood? Field studies have suggested that CH_4_ could originate in the soil as they report high CH_4_ concentrations in deep soil close to the measured stem^21^, a decrease in CH_4_ emissions with stem height^18^, or correlations between stem and soil CH_4_ fluxes^20^. In contrast, high CH_4_ concentrations in the heartwood^34,35^, correlations between heartwood CH_4_ concentrations or heartwood water content and stem emissions^19^, or the lack of relationships between stem emissions and soil variables^12,17^ suggest that emitted CH_4_ could also be produced in the heartwood by the activity of methanogenic Archaea and/or heart rot infection. Additionally, wood decomposition by basidiomycete fungi and stress-induced (e.g. UV light, temperature, insect herbivory) degradation of methoxyl groups in pectin or lignin are also possible sources of locally produced CH_4_ within tree stems^36–38^. Some studies report results consistent with CH_4_ production both in the soil and in the heartwood^17^. The mechanisms underlying stem N_2_O emissions are even less known, and consequently, the origin of stem CH_4_ and N_2_O emissions is still a topic of ongoing research.

A third issue is the interest for upscaling stem CH_4_ and N_2_O fluxes to quantify their role in ecosystem GHG balance. Studies reporting upscaled stem CH_4_^8,12,20^ and N_2_O emissions^2,20,32,39^ are subjected to multiple uncertainties that need to be addressed to obtain accurate ecosystem-level GHG fluxes^33^. The magnitude of GHG emissions vary spatially, within individual tree stems, between stems of different diameters (e.g., stem *vs* twigs) and among stems within a forest stand. Moreover, the characterization of seasonal variability of stem GHG emissions is usually based on sporadic manual measurements, which misrepresent information about pulses and daily variability^40^. To our knowledge, only one study reports limited information on sub-daily co-located measurements of CO_2_ and CH_4_ stem emissions^17^ and suggests that stem CH_4_ emissions could show a daily pattern (with only 3 days of measurements). If a diurnal pattern is consistent across tree stems and throughout the growing season, then manual measurements may bias estimates of daily total emissions^33^ and therefore the net impact of stem CH_4_ emissions for the ecosystem carbon balance. To our knowledge, there is no information based on automated measurements of stem N_2_O fluxes.

Here, we implement an automated system to continuously measure (i.e., 1-hour resolution) CO_2_, CH_4_ and N_2_O fluxes from stems and soils in an upland forested area in order to better describe magnitudes, emissions and origin of CH_4_ emissions. We measured these three GHGs at two stem heights (75 and 150 cm) in a bitternut hickory (*Carya cordiformis* (Wangenh.) K.Koch), and at the adjacent soil. We postulate that high temporal frequency measurements provide: (a) unprecedented estimates of magnitudes and patterns of CO_2_, CH_4_ and N_2_O stem fluxes; and (b) insights about temporal correlations and potential sources of GHG stem fluxes. We highlight that technical and scientific advances are needed to better understand the underlying mechanisms for GHG stem emissions, their incorporation in process-based models, and to quantify their role in local-to-global GHG budgets.

## Results

**Figure 1:**
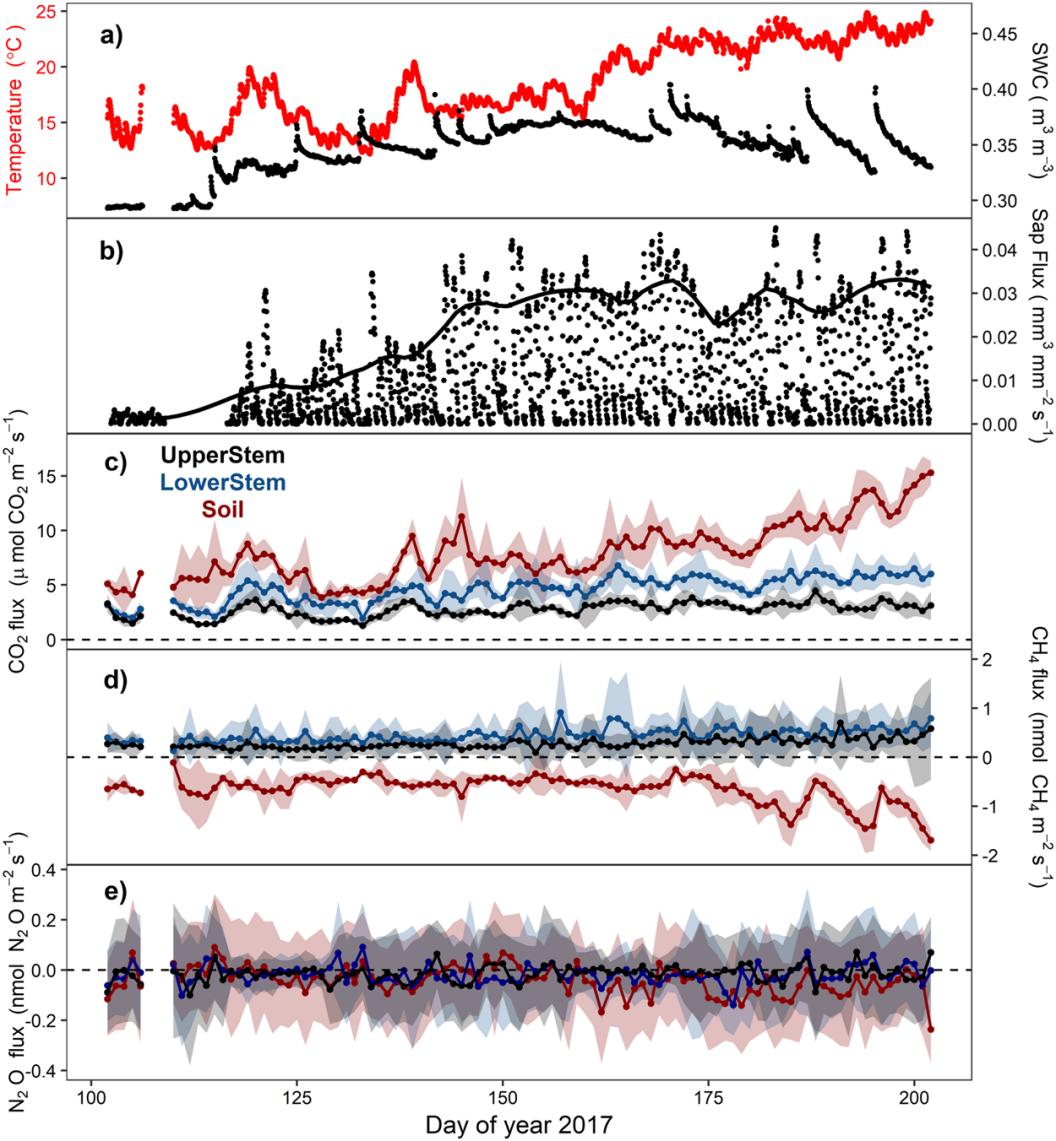
Seasonal course of hourly mean soil temperature and soil water content (SWC) (panel (**a**)), sap flux per unit sapwood area (SF) (panel (**b**)) and daily means of CO_2_, CH_4_ and N_2_O fluxes (panels (**c–e**), respectively; mean ± SD) associated to UpperStem (black), LowerStem (blue) and Soil (red) chambers. Line in panel b) depicts smoothed patterns for midday SF values.

Over the study period, the tree stem acted as a net source of CO_2_ and CH_4_ but a sink of N_2_O. The adjacent soil was a net CO_2_ source but a CH_4_ and N_2_O sink (Table 1). For CO_2_, emissions were higher in the soil and decreased with height in the tree stem, being 8.24 ± 0.55, 4.76 ± 0.22 and 2.83 ± 0.13 µmol m^−2^ s^−1^ on average for Soil, LowerStem and UpperStem, respectively. CH_4_ emissions also decreased with height along the tree stem (0.46 ± 0.03 and 0.28 ± 0.02 nmol m^−2^ s^−1^ for LowerStem and UpperStem, respectively), but the soil was a clear sink of this gas (−0.66 ± 0.06 nmol m^−2^ s^−1^). CO_2_ and CH_4_ emissions in LowerStem were consistently higher than UpperStem emissions over the study period (Fig. 1). Mean N_2_O uptake was higher in soil (−0.046 ± 0.011 nmol m^−2^ s^−1^) and decreased with stem height (−0.017 ± 0.008 and −0.014 ± 0.006 nmol m^−2^ s^−1^ for LowerStem and UpperStem, respectively).

**Table 1:** CO_2_, CH_4_ and N_2_O mean fluxes and cumulative fluxes over the study period (mean ± 95% CI) for each position (UpperStem, LowerStem and Soil).

	CO_2_		CH_4_		N_2_O	
	*mean*	*cumulative*	*mean*	*cumulative*	*mean*	*cumulative*
	µmol CO_2_ m^−2^ s^−1^	kg CO_2_ m^−2^	nmol CH_4_ m^−2^ s^−1^	g CH_4_ m^−2^	nmol N_2_O m^−2^ s^−1^	g N_2_O m^−2^
UpperStem	2.83 ± 0.13	0.39 ± 0.05	0.28 ± 0.02	0.106 ± 0.008	−0.014 ± 0.006	−0.005 ± 0.002
LowerStem	4.76 ± 0.22	0.66 ± 0.08	0.46 ± 0.03	0.175 ± 0.011	−0.017 ± 0.008	−0.006 ± 0.003
Soil	8.24 ± 0.55	1.14 ± 0.21	−0.66 ± 0.06	−0.251 ± 0.023	−0.046 ± 0.011	−0.017 ± 0.004

**Figure 2:**
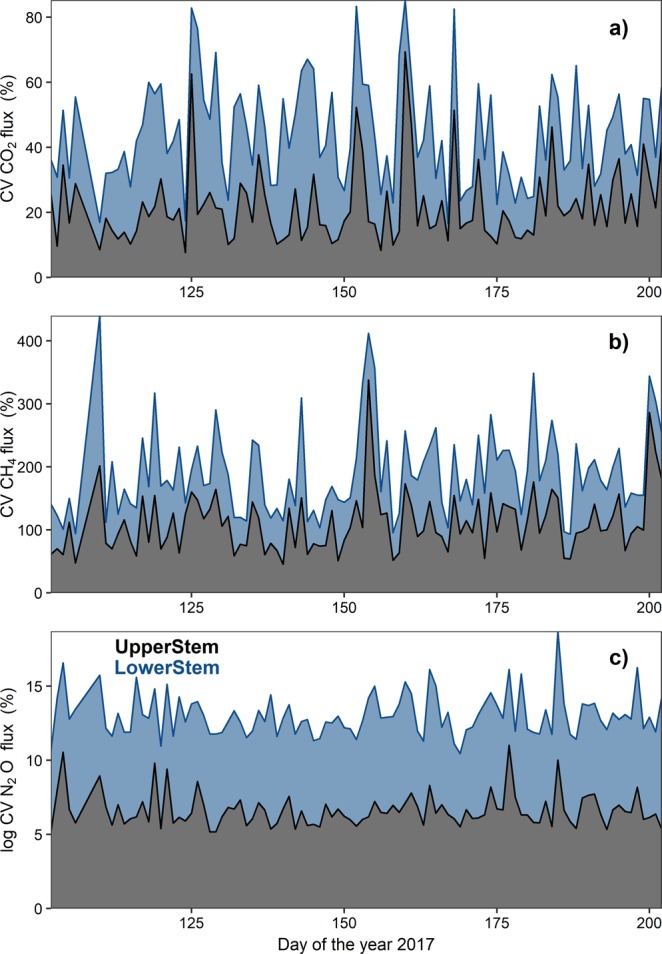
Seasonal course of the daily coefficient of variation (CV) of CO_2_, CH_4_ and N_2_O fluxes (panels (**a–c**), respectively) associated to UpperStem (black), LowerStem (blue) chambers. CV is reported in absolute values. CV is log transformed for N_2_O.

Overall, CO_2_ and CH_4_ stem emissions increased over the growing season in parallel with soil temperature and SF (Fig. 1c,d). Both GHGs showed similar seasonal patterns between UpperStem and LowerStem. In contrast, stem N_2_O fluxes did not show any clear seasonal pattern or similarity between UpperStem and LowerStem. Like stem CO_2_ emissions, soil CO_2_ emissions increased along the growing season. However, soil CH_4_ flux showed the opposite pattern than stem CH_4_, whereby soils showed an increasing CH_4_ uptake over the growing season. Soil N_2_O fluxes showed a slight increase in uptake (i.e., fluxes more negative) during the growing season (Fig. 1c). Despite seasonal patterns were evident for CO_2_ and CH_4_ fluxes, the high-temporal resolution data revealed a high variability in the magnitude of the fluxes (Fig. 2, SFig. 1).

**Figure 3:**
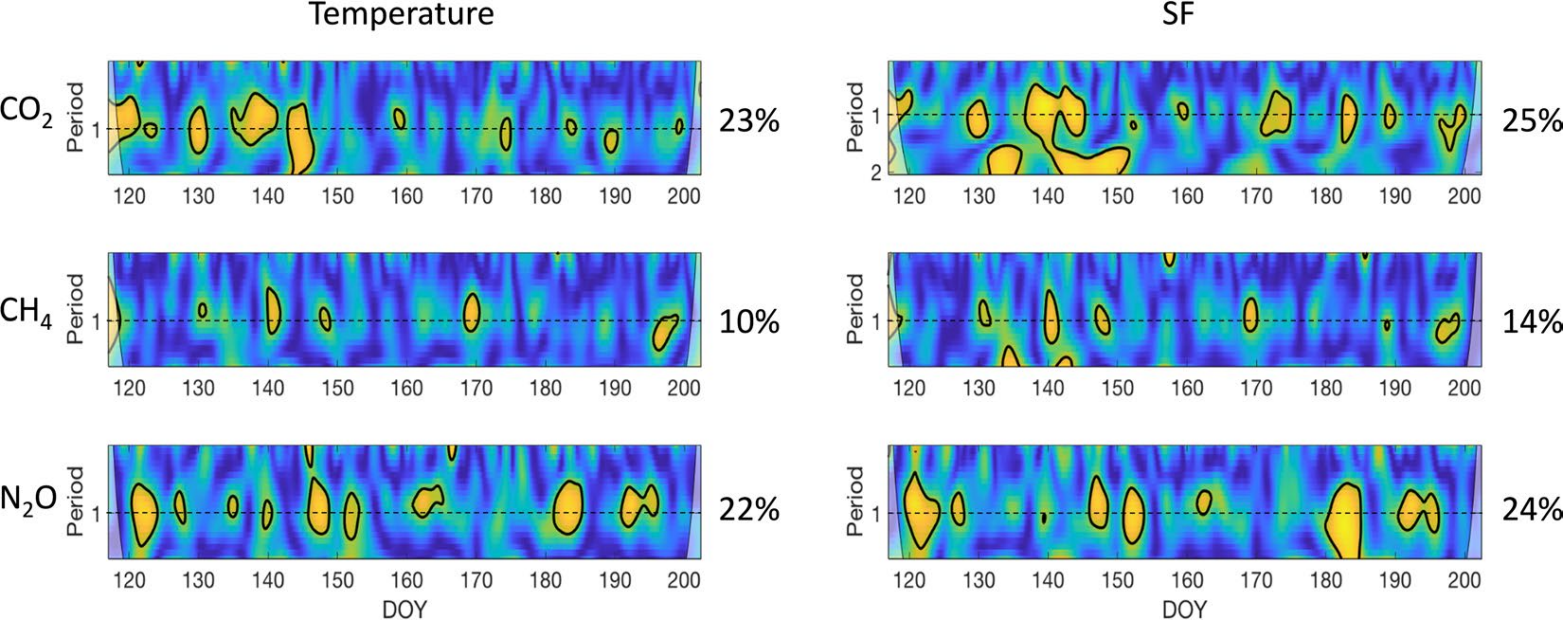
Wavelet coherence analyses output and percentage of days with daily significant correlations between CO_2_, CH_4_ and N_2_O measures at LowerStem with Temperature (left panels) and SF (right panels) from hourly data. Yellow color indicates significant temporal correlations (p < 0.05).

Stem CO_2_, CH_4_ and N_2_O fluxes showed temporal correlations at the 1-day period with temperature or SF (Fig. 3). However, these temporal correlations were not consistent throughout the growing season. Stem CO_2_ and N_2_O fluxes showed temporal correlations at the 1-day period with temperature (23% of the days) and SF (25% of the days) (similar percentage for both gases). In contrast, stem CH_4_ fluxes showed temporal diurnal correlations at the 1-day period with temperature (10% of the days) and SF (14% of the days). Overall, diurnal correlations between GHG fluxes and their drivers did not have a relationship with the temporal progression of the growing season. In other words, there were no differences in temporal correlations at the 1-day period between the early and late growing season.

**Figure 4:**
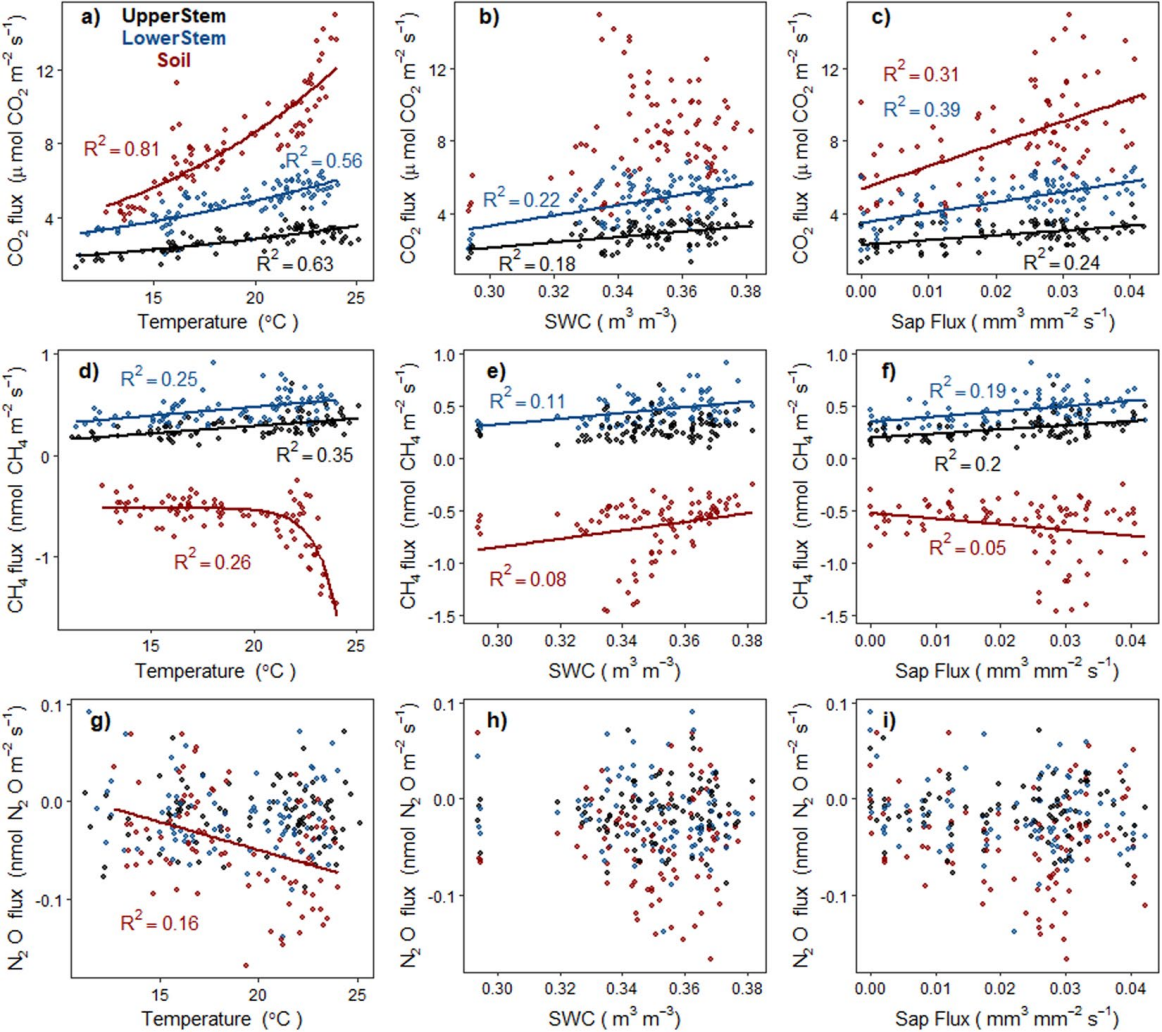
Daily CO_2_, CH_4_ and N_2_O regressions with temperature, soil water content and sap flux for UpperStem, LowerStem and Soil (black, blue and red, respectively). Soil temperature was used for soil emissions comparisons and stem temperature measured at each height was used for stem emissions comparisons. Exponential regression was fitted for CO_2_ and temperature (panel a), sigmoidal regression was fit for soil CH_4_ and temperature (panel d) and linear regressions were fitted for the other cases. Regression fit and R^2^ were placed if significant (p < 0.05).

Daily-mean GHG fluxes from the three locations correlated with temperature, SWC and SF, especially CO_2_ and CH_4_ (Fig. 4). However, GLS models showed that interactions between temperature, SWC and SF (rather than each independent variable) explain a large fraction of the variability of seasonal GHG emissions (Table 2). For example, soil CH_4_ fluxes showed a correlation of 0.26 with temperature and 0.08 with SWC, but their interaction could explain 92% of their temporal variability. Best models for CO_2_ fluxes across different locations were similar. First order interactions between temperature, SWC and SF could explain >90% of the seasonal variability of CO_2_ emissions (Table 2). Models explaining stem seasonal CH_4_ fluxes were similar at the two heights. Stem CH_4_ fluxes were explained by temperature and SF in UpperStem and by temperature and SWC in LowerStem, accounting for 40 and 33% of the seasonal variability, respectively. In the soil, the interaction between temperature and SWC had a much stronger effect on CH_4_ fluxes than in the tree stem, explaining up to 92% of seasonal variability (Table 2). Stem and soil N_2_O fluxes were less explained by environmental drivers. For UpperStem, the independent effects of temperature, SWC and SF were included in the best model, but only explained 10% of the variability of N_2_O fluxes. For soil, only temperature affected N_2_O fluxes, explaining 22% of the seasonal variability.

**Table 2:** Summary of the selected models for each greenhouse gas (CO_2_, CH_4_ and N_2_O) and each position (UpperStem, LowerStem and Soil). All variables were scaled to improve the performance and interpretability of the models.

MODEL	Variables	Estimate	SE	t-value	p-value
UpperStem logCO_2_	(Intercept)	−0.113	0.125	−0.909	0.366
adjR^2^ = 0.93	Temperature	0.634	0.084	7.578	<0.001
p-value < 0.001	SWC	0.566	0.106	5.351	<0.001
	SF	−0.001	0.058	−0.002	0.998
	Temp*SF	−0.200	0.045	−4.456	<0.001
	SWC*SF	0.318	0.067	4.772	<0.001
LowerStem logCO_2_	(Intercept)	0.106	0.142	0.747	0.457
adjR^2^ = 0.92	Temperature	0.517	0.095	5.446	<0.001
p-value < 0.001	SWC	0.192	0.104	1.841	0.069
	SF	0.159	0.049	3.266	0.002
	Temp*SWC	0.206	0.079	2.606	0.011
	Temp*SF	−0.254	0.044	−5.723	<0.001
Soil logCO_2_	(Intercept)	0.162	0.254	0.639	0.525
adjR^2^ = 0.99	Temperature	1.011	0.116	8.684	<0.001
p-value < 0.001	SWC	0.174	0.095	1.825	0.072
	SF	0.081	0.036	2.255	0.027
	Temp*SWC	−0.240	0.072	−3.315	0.001
UpperStem CH_4_	(Intercept)	0.001	0.088	0.010	0.992
adjR^2^ = 0.40	Temperature	0.451	0.098	4.488	<0.001
p-value < 0.001	SF	0.254	0.097	2.620	0.01
LowerStem CH_4_	(Intercept)	0.002	0.096	0.021	0.983
adjR^2^ = 0.33	Temperature	0.447	0.097	4.613	<0.001
p-value < 0.001	SWC	0.279	0.096	2.903	0.005
Soil CH_4_	(Intercept)	−0.142	0.090	−1.580	0.118
adjR^2^ = 0.92	Temperature	−0.666	0.085	−7.822	<0.001
p-value < 0.001	SWC	0.761	0.076	9.966	<0.001
	Temp*SWC	0.427	0.065	6.540	<0.001
UpperStem N_2_O	(Intercept)	0.003	0.114	0.026	0.980
adjR^2^ = 0.10	Temperature	0.240	0.126	1.899	0.061
p-value = 0.032	SWC	0.260	0.136	1.923	0.058
	SF	−0.404	0.143	−2.816	0.006
LowerStem N_2_O					
p-value = n.s.					
Soil N_2_O	(Intercept)	−0.006	0.131	−0.045	0.964
adjR^2^ = 0.22	Temperature	−0.382	0.129	−2.960	0.004
p-value = 0.001					

**Figure 5:**
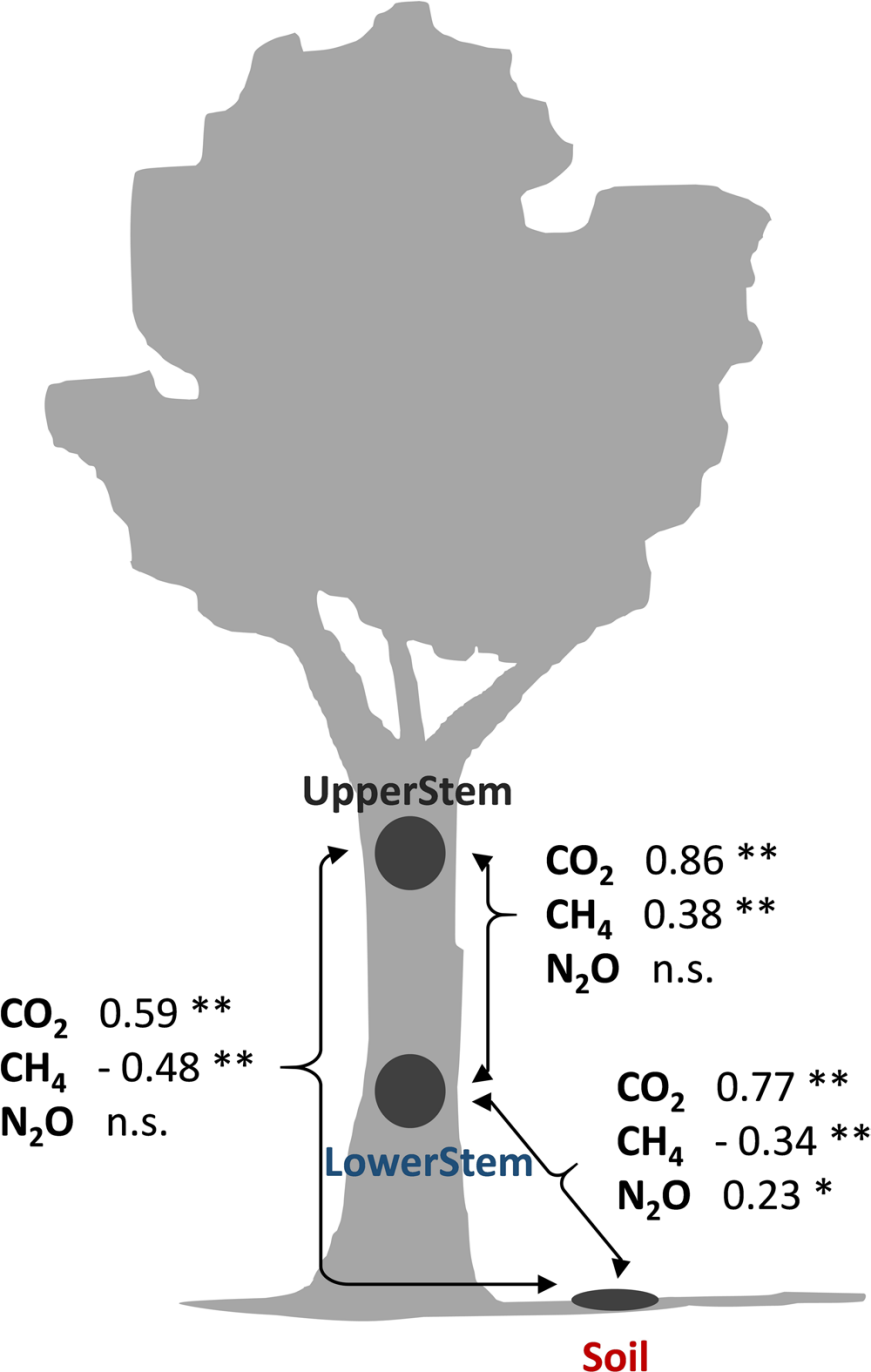
Correlation between daily-mean CO_2_, CH_4_ and N_2_O fluxes between different locations within the tree (Pearson correlation; **p-value < 0.001, *p-value < 0.05).

Among locations, CO_2_ emissions were positively correlated, with stronger relationships between UpperStem and LowerStem, intermediate relationships between LowerStem and Soil and the least correlation between UpperStem and Soil (Fig. 5). CH_4_ fluxes were well correlated among locations, with a positive relationship between UpperStem and LowerStem and negative relationship between stem and soil emissions. We only found a positive correlation between LowerStem and Soil N_2_O fluxes (Fig. 5).

**Figure 6:**
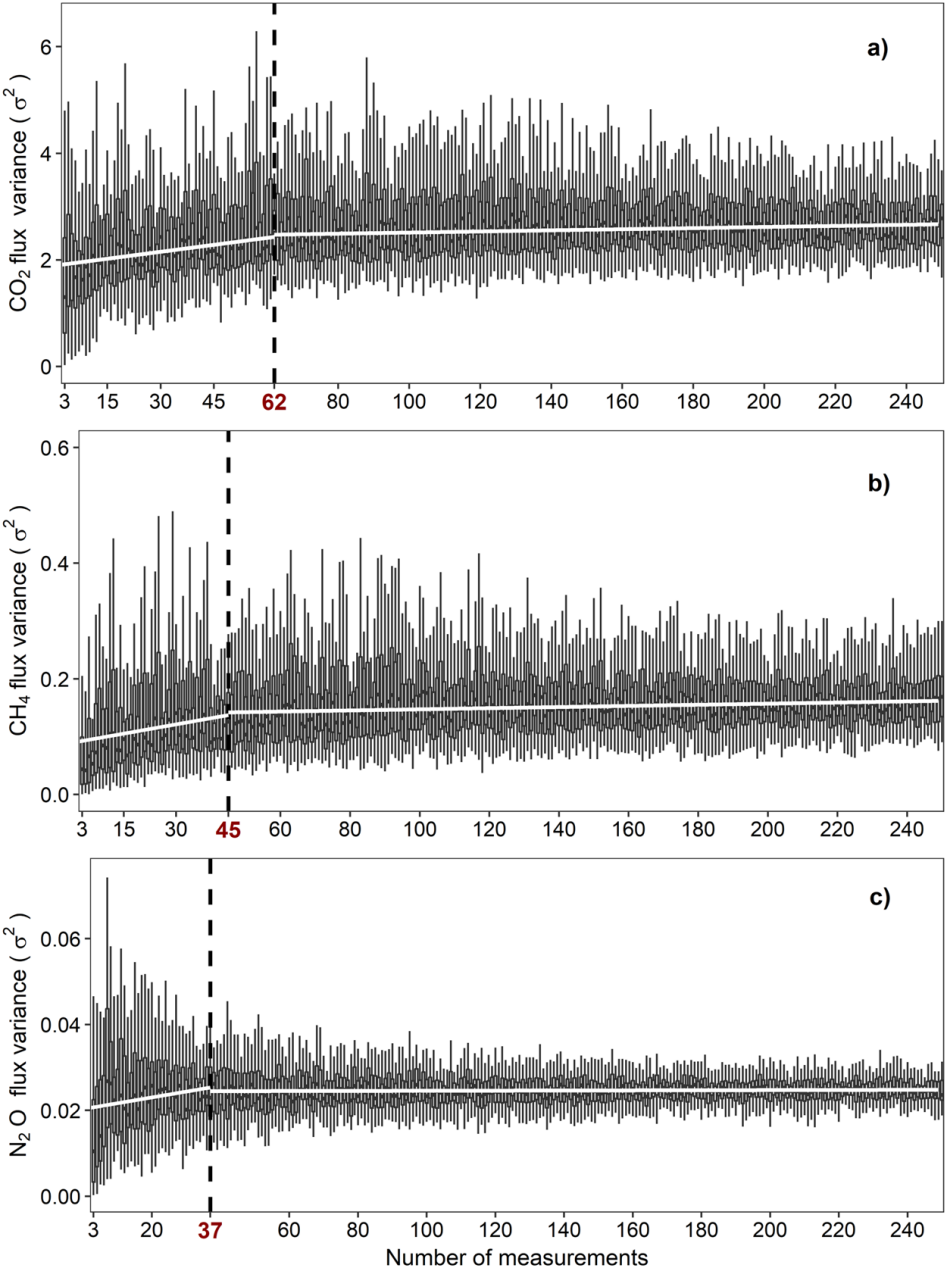
Minimum number of measurements for estimating the whole period stem fluxes, represented by the breakpoint between two variance regression trends (white solid lines). Median, quartiles and data range of CO_2_, CH_4_ and N_2_O (panels (**a–c**), respectively) are represented in box-and-whisker plots as a function of sample size. σ is expressed in µmol m^−2^ s^−1^ for CO_2_ and in nmol m^−2^ s^−1^ for CH_4_ and N_2_O.

Our results show that that stem GHG fluxes in a tree from an upland forest are highly variable in time (Figs 1, 2). That said, an open question is to know how many measurements are needed for estimating seasonal stem GHG emissions. Our analysis based on random resampling of high-frequency measurements from the total pool of 5000 measurements for each GHG showed that the minimum number of punctual measurements required for estimating seasonal stem GHG emissions (based on variance stabilization) was 62, 45 and 37 for CO_2_, CH_4_ and N_2_O, respectively (Fig. 6). In other words, these are the minimum number of measurements necessary to explain the same amount of variance as using the whole population of measurements.

## Discussion

Our results show that tree stems and soils were active surfaces for exchange for GHGs with the atmosphere at multiple time scales. Stems were net sources of CO_2_ and mean efflux values (3.80 ± 0.18 µmol m^−2^ s^−1^) where similar^41,42^ or higher^12,21,43^ than those reported in previous studies from upland forests. The soil was also a net source of CO_2_ with a mean flux of 8.24 ± 0.55 µmol m^−2^ s^−1^, consistent with the growing season average derived from autochambers at the study site (8.54 µmol m^−2^ s^−1^)^44^, but higher than the efflux reported in 99% of studies from temperate ecosystems worldwide^45^, indicating that the study site is a hot spot for soil respiration. Soils were net CH_4_ sinks (−0.66 ± 0.06 nmol m^−2^ s^−1^), with values half of those reported for other temperate forests^12,16,21^. However, despite this soil net uptake, stems were a net source of CH_4_ (mean emissions from both heights was 0.37 ± 0.18 nmol m^−2^ s^−1^). These CH_4_ stem emissions were within the range reported in nearby temperate forests^12,17^, but the literature reports a wide range, from close to zero emissions^20,23^ to 6-fold in our findings^19^. On average, stems were a N_2_O sink during the study period (−0.016 ± 0.008 nmol m^−2^ s^−1^) and soils were almost three times a stronger sink (−0.045 ± 0.011 nmol m^−2^ s^−1^). There is limited information to compare stem N_2_O fluxes, but a few studies using manual measurements report that stems could act as a N_2_O source^15,20,22,32,39^ or sink^30^. Our results revealed that there is a large temporal variability in stem N_2_O emissions, so manual measurements could lead to biased daily and seasonal estimates due to the limited sampling rate as has seen in soils^40^ or for other gases^33^. Soils were net sinks of N_2_O, and despite they are commonly considered as net sources in upland forests, there are numerous reports of soil N_2_O uptake in forests ecosystems^28^. Our results clearly suggest that tree stem emissions (and not only soils) could play a crucial role for the ecosystem-scale (e.g., a temperate forest) GHG balance.

Stem emissions showed temporal patterns from daily- (within days) (Fig. 3) to seasonal-scales (Fig. 1) for the three GHGs. At daily-scales, emissions showed high variability (Fig. 2, SFig. 1), especially for CH_4_ and N_2_O. We did not find temporal clusters in flux variability throughout our study (e.g., dry periods could have lower emissions variability than wet periods), but longer studies of high-frequency measurements might be useful for detecting potential temporal changes in emissions variability. We found temporal correlation at the 1-day time-period for specific days between GHGs and temperature or SF (more frequent for CO_2_ or N_2_O than for CH_4_ fluxes). Several studies have also shown that diurnal patterns of stem CO_2_ emissions are correlated with temperature or SF^41,46^. Stem CH_4_ fluxes showed a temporal correlation at the 1-day time period with temperature and SF over 10 and 14% of the studied period, respectively. Our study demonstrates temporal CH_4_ emissions were associated with SF at diurnal (Fig. 3) and seasonal scales (Fig. 4, Table 2). One study reporting three days of CH_4_ measurements from tree stems using autochambers found one tree stem (*Liriodendron tulipifera*) with daily cycles, but no daily cycles were observed in a second tree stem (*Fagus grandifolia*)^17^. This lack of consistency between individual trees suggest that more information derived from automated measurements is needed to clarify patterns among tree species and individuals. Our results show that stem CH_4_ emissions can present daily cycles but these are not consistent throughout the growing season. In fact, persistent daily cycles may be the exception rather than the norm for CH_4_ (and N_2_O) emissions from stems in upland forests. To our knowledge, there are no published high-frequency measurements of tree stem N_2_O fluxes. Our results show that over 25% of the studied period, stem N_2_O uptake showed a temporal correlation with temperature and SF at the 1-day time period (Fig. 3). Even if GHGs production had a clear and consistent diurnal patterns, the low radial gas diffusivity in stems^47,48^ would smooth the potential daily pattern of the emissions at the stem-surface level. This would be consistent with the lack of daily cycles observed for most of the studied period.

Seasonal patterns of stem GHGs were evident for CO_2_ and CH_4_ (Fig. 1). Both stem heights and soil showed similar CO_2_ seasonal patterns, explained by interactions between temperature, SWC and SF (Fig. 4, Table 2). High SWC under warm temperatures could enhance soil activity (both autotrophic and heterotrophic components) resulting in high soil respiration^49–51^, promoting sap flux and increasing stem respiration^41,52^. Seasonal CH_4_ patterns were also similar for both stem heights, with temperature, SF and SWC as controlling drivers. Seasonal stem CH_4_ emissions with temperature dependency were also found in another temperate forest^19^, but not detected in other upland forests nearby our study site^12,16^.

We have demonstrated that automated chamber-based measurements are a powerful tool to properly estimate seasonal or daily stem emissions and to study temporal correlations and drivers of soil-tree GHG fluxes. It is evident that multiple measurements per day are required for studying diurnal patterns of stem CH_4_ emissions^33^ given the high diurnal variability of the emissions (Fig. 2), but we also demonstrated that they could be crucial for studying seasonal dynamics and long-term mean magnitudes. Manual measurements of stem emissions cannot capture the high-variability of stem emissions at both diurnal and seasonal scales^33^, with implications for the estimation of temporal trends and GHG budgets. Using automated measurements, we calculated that the minimum number of random measurements required for properly estimating the whole period stem CH_4_ emissions was 45 measurements (Fig. 6). Multiple measurements per day could also better detect seasonal patterns than single manual measurements, integrating the large short-term heterogeneity of stem emissions as seen in soils^53,54^ or integrating diurnal patterns^33,55^. The ability to detect seasonal patterns in our dataset, but not in datasets with much less frequent sampling (e.g., once every 2–4 weeks)^12,16,22^, suggests that future studies may need to incorporate more frequent sampling in order to detect temporal patterns against a background of high variability in flux rates. The need for high-frequency measurements could be even more important for stem N_2_O emissions, since the magnitude of these emissions is low and the short-term variability is high (Fig. 2) (quickly shifting between positive and negative fluxes within hours; SFig. 1).

Our results support the general consensus that CO_2_ emitted by stems could be partially produced belowground^9^. First, emissions were higher in the soil and decreased with stem height, which is consistent with a belowground origin and subsequent stem degasification. Second, if CO_2_ is originated belowground, it should be partially dissolved into soil water and transported through the sap; consequently, we found correlations between stem emissions and SWC of SF at diurnal and seasonal scales. Third, stem and soil showed similar seasonal patterns with high correlation between soil and stem emissions, which suggest that both fluxes may depend on soil CO_2_ production.

There is no scientific consensus regarding the origin of CH_4_ emitted by the stems^13^, but our findings suggest a potential belowground origin. First, we found higher CH_4_ emissions in LowerStem than in UpperStem as observed in other studies^17,21^, which can be interpreted as stem degasification with height. Belowground processes could regulate the origin of CH_4_ emitted by stems regardless of soil being a sink of CH_4_. Soils can be net CH_4_ sinks at surface level but could produce CH_4_ at deeper depths^21^. We postulate that it is possible that tree roots might take up water from deep layers with dissolved CH_4_ produced in deep anoxic layers or anoxic microsites^56,57^ and bypass the surface methanotrophic layers in the soil^7^. Second, across certain days along the growing season we found temporal correlation at the 1-day period between CH_4_ fluxes and SF, which directly links stem emissions with stem water transport and thus, with water coming from belowground in the transpiration stream. As mentioned before, the lack of correlation at 1-day period for most of the growing season could be explained by the low radial stem diffusivity, but this limitation is likely less important at seasonal scale. Therefore, we found strong correlation between seasonal CH_4_ stem emissions and SF or SWC, indicating that belowground could be the origin of CH_4_ during the growing season. However, we also found a negative correlation between CH_4_ stem emissions and soil uptake. Stem emissions and soil uptake could be related by sharing belowground biochemical pathways between CH_4_ production and consumption across the soil profile. Under low soil moisture conditions, we would expect low transpiration and thus, less stem CH_4_ emissions, but also high soil diffusivity resulting in higher diffusion of atmospheric CH_4_ and O_2_ into the soil, and consequently more soil CH_4_ consumption. On the other hand, high SWC would enhance transpiration, resulting in higher sap flux and high stem emissions but it would also cause a reduction of soil diffusivity, then a reduction in soil oxygenation and CH_4_ diffusion from the atmosphere and consequently a reduction of CH_4_ soil uptake^58,59^.

Although our data support a possible soil origin of stem CH_4_ emissions, we recognize that there is an ongoing debate about underlying mechanisms^13,33^. Findings from other studies such as internal heartwood CH_4_ production^18,60^, high heartwood CH_4_ concentrations^34,35^, correlation between CH_4_ stem emissions and moisture CH_4_ concentration in heartwood^19^, or the presence of methanogenic archaea inhabiting the heartwood^61^ would suggest that emitted CH_4_ could also be produced in the stems. Observational studies measuring simultaneous high-frequency fluxes of stems and soils coupled with soil and heartwood CH_4_ concentrations, the analysis of heartwood microbial composition and isotopic experiments tracing the origin of the emitted CH_4_ would shed some light on this debate^33^.

N_2_O stem emissions were low and highly variable, making our results challenging to interpret as have been reported for ecosystem-scale N_2_O fluxes^62^. We found a significant correlation between soil and LowerStem N_2_O, but not with UpperStem, which could mean that soil influence is decreasing with stem height. Other studies have found a correlation between soil and stem emissions in upland forests under natural conditions^20^ or after soil fertilization^32^, suggesting that N_2_O emitted by stems could be originated belowground, but those studies reported emissions and not uptake. In our case, we speculate that stem and soil fluxes may not be directly related to stem water transport as for CH_4_ but indirectly, sharing drivers that potentially promote N_2_O consumption in stems and soils. This could be supported by the fact that temperature, SF and SWC explained part of the variability of both, soil and LowerStem N_2_O uptake. Stem N_2_O consumption has been described in the presence of cryptogamic cover^30^, but the apparent absence of this kind of covers in our tree makes this explanation unlikely. An alternative explanation for this N_2_O consumption could be heartwood decomposition. Net consumption of N_2_O and production of CH_4_ could be indicators of anaerobic wood decay as shown for deadwood^63^, which agrees with the observed CH_4_ emissions and N_2_O uptake in stems. Further understanding of these processes would require high-frequency CH_4_ and N_2_O emissions coupled with measurements of heartwood decay and presence of denitrifying and methanogenic bacteria. Finally, if sap has lower N_2_O concentrations than the atmosphere (which could be expected since soils are net sinks of N_2_O), diffusion of N_2_O from the atmosphere into the sapwood driven by this concentration gradient might result in a net N_2_O uptake. The positive correlation between LowerStem and Soil fluxes might be consistent with this explanation.

We highlight that CH_4_ and N_2_O emissions from trees are an emerging science frontier for plant physiology with implications for ecosystem processes, ecosystem management and atmospheric sciences. In upland forests, adequate characterization of temporal variability in CH_4_ and N_2_O emissions from stems and soils may be even more important than accounting for spatial variability^20,33^. The high temporal variability of stem GHG emissions highlight the need for measuring tree stem emissions with high temporal frequency and for longer periods in order to understand the multi-temporal dynamics and to better characterize the magnitude of these stem GHG fluxes. Information of temporal variability of stem GHG emissions obtained with automated measurements could therefore be useful for designing spatial experiments using manual measurements. Consequently, these and future studies will continue to provide insights about drivers and will aid to formulate and parameterize process-based ecosystem models that will include CH_4_ and N_2_O fluxes from tree stems.

## Materials and Methods

### Study site

We performed this study in a temperate forested area in the Mid Atlantic of the USA (St Jones Estuarine Reserve, a component of the Delaware National Estuarine Research Reserve [DNERR] [39°5′20′′N, 75°26′21′′W]). Mean annual temperature and precipitation were 13.3 °C and 1119 mm, respectively. Soils are Othello silt loam with a texture of 40% sand, 48% silt and 12% clay, and with 5.82%, 0.39% and 577.05 mg Kg^−1^ of total C, total N and total P, respectively. The forested site is dominated by bitternut hickory (*Carya cordiformis*), American holly (*Ilex opaca* (Ashe)), black gum (*Nyssa sylvatica* (Marshall)), eastern red cedar (*Juniperus virginiana* L.) and sweet gum (*Liquidambar styraciflua* L.). See previous study for more information related to the study area^44^.

### Experimental setup

We continuously measured CO_2_, CH_4_ and N_2_O fluxes (1-hour resolution) from April to July 2017 (100 days) around a hickory tree (diameter at breast height [DBH] of 51 cm and 14 m height) at three different locations: (a) two stem heights represented as UpperStem (150 cm) and LowerStem (75 cm); and (b) one adjacent soil (1.5 m from the stem base). We installed two PVC collars (317.8 cm^2^) at the respective heights on the stem surface of the tree, and inserted a third collar 5 cm into the soil. Automated chambers (Li-COR 8100-104, Lincoln, Nebraska), controlled by a multiplexer (Li-COR 8150, Lincoln, Nebraska), were installed to measure fluxes at each collar. Since the automated chambers are designed to be placed horizontally to measure soil fluxes, we modified the stem-surface chambers to perform measurements in a vertical position. Automated measurements were taken with a closed-path infrared gas analyzer (Li-8100A, Lincoln, Nebraska) coupled to a cavity ring-down spectrometer (Picarro G2508, Santa Clara, California) as described in previous publications^44,64^. For each flux observation, gas concentrations were measured every second during 150 and 350 seconds for soil and stem chambers, respectively. Co-located with the chambers, we installed soil temperature and volumetric soil moisture (SWC) sensors at 10 cm depth into the soil and temperature sensors at 5 cm into the stem (EC-5, Decagon Devices, Pullman, WA).

We measured sap flow density (SF, mm s^−1^) every 15 min from April to July 2017 (at 150 cm height; 100 days), using constant heat dissipation sensors^65^ manufactured in our laboratory. Probe length was 1 cm to minimize the effect of high-radial gradient of sap flow density, which is common for species with ring-porous xylem anatomy such as hickory^66^. We inserted probe pairs into the xylem with a vertical separation of 12 cm and covered with reflective bubble wrap to minimize natural temperature gradients. Sap flow density was calculated using the original calibration^65^, considering zero flow conditions only during nights with low evaporative demand and stable sensor readings^67^. Meteorological and soil variables were recorded at 15-minutes intervals (measured every second during a 1-min period and subsequently averaged to 15-minutes) using a digital data logger (Em50, METER Group, Pullman, WA). The variables were SWC, soil temperature (5TE, METER Group, Pullman, WA), air relative humidity, air temperature, atmospheric pressure (VP-4 Sensor (Temp/RH/Barometer), METER Group, Pullman, WA) and wind speed and direction (DS-2, METER Group, Pullman, WA). There was a gap for GHG measurements between April 16th and 20th and for SF measurements between April 19th and 27th due to power failure.

### Greenhouse gas fluxes

We calculated CO_2_, CH_4_ and N_2_O fluxes from the raw data collected by the Picarro G2508 using Soil Flux Pro Software (v4.0; Li-COR, Lincoln, Nebraska). For CO_2_ and CH_4_, both linear and exponential fits were adjusted to the measurements of concentrations of each gas and the fit with higher R^2^ was kept to calculate the fluxes^44^. Then, we applied a quality assurance/quality control (QA/QC) based on R^2^ values of measurements. When the R^2^ of the calculated CO_2_ efflux was lower than 0.9 (297 of 7265 measurements; 4% of the data) we considered that the physical conditions inside the chamber were not appropriate to calculate accurate fluxes (likely due to an improper chamber closure). Consequently, calculations were removed for CO_2_, CH_4_ and N_2_O fluxes for that particular time-stamp and replaced as not-a-number (i.e, NaN). When conditions inside the chamber were appropriate (R^2^ for CO_2_ > 0.9), we kept the fluxes for the three gases regardless of R^2^ of CH_4_ and N_2_O. For N_2_O measurements, we only calculated fluxes using a linear fit to avoid bias induced by applying an exponential fit at near-zero or negative fluxes^68^; we highlight that most N_2_O fluxes were close to zero.

### Statistical analyses

For studying temporal correlations at diurnal time-scale (i.e., 1-day period) between stem GHG emissions and temperature or SF, we applied wavelet coherence analyses (WCA) for each GHG using hourly LowerStem emissions. We highlight that emissions from both LowerStem and UpperStem showed similar temporal patterns and consequently provide similar results in a time series analysis using the frequency domain. WCA measures transient signals or signals whose amplitude varies with time between two time series^69,70^. This technique has been applied for analyzing ecosystem-scale fluxes^71,72^, for soil respiration^73,74^, and for studying the relation between soil CO_2_ and SF fluxes^75^. We assessed the statistical significance of common power between the two time series (0.05 significance level) applying 10,000 Monte Carlo simulations of white noise time^69^. Finally, for each pair of variables we calculated the percent of days when WCA showed significant temporal correlations at the 1-day period. WCA was not applied with SWC since no diurnal pattern was expected for SWC^75^. Additionally, we calculated the daily coefficient of variation for each gas throughout the experiment.

We calculated linear correlations between CO_2_, CH_4_ and N_2_O fluxes and temperature, SWC and SF, controlling for leverage points and Cook’s distance. We used exponential and sigmoidal regressions for CO_2_ and temperature, and soil CH_4_ and temperature, respectively, to achieve better regression fit.

Additionally, we applied generalized least squares linear models (GLS) for studying seasonal relationship between stem emissions and stem temperature, SWC and SF. We fitted one model for each GHG using daily means for temperature and SWC, and midday measurements of SF (averaged between 11 and 13 h), as an indicator of maximum stem water transport rates within a day. We log-transformed CO_2_ efflux values in order to linearize its relation with temperature, and scaled all variables to improve the performance and interpretability of the output models^76^. We tested the variance inflation factor (VIF) to assess predictor collinearity between temperature, SWC and SF, but in all cases VIF was lower than two, indicating low collinearity^77^. Assessing for VIC also controls for potential confounding factors associated with the hierarchical controls of temperature and SWC on SF. Additionally, we included the correlation structure in the models in order to avoid temporal autocorrelation between measurements. For the model selection, we evaluated all possible models combining temperature, SWC, SF and their first order interactions or each GHG and location (i.e., UpperStem, LowerStem and Soil) in order to achieve the minimum adequate model according to corrected Akaike information criterion (AICc). When two models were not statistically different (p > 0.05 when performing a likelihood ratio tests between models differing less than two AICc units), we kept the more complex one in terms of variables and number of interactions, since our aim was to understand the relation between gas emissions and their potential drivers rather than create a predictive model. Summaries of all models differing less than two AICc units from the selected model are presented in S.Table 1. Once we had the minimum adequate models, we calculated the adjusted R^2^.

We calculated the minimum number of stem measurements required for estimating the whole-period average per each gas using a resampling routine with an increasing number of measurements (n) (from n = 3 to n = 250 measurements, with replacement)^78^. For each *n*, 40 replicates were obtained by randomly selecting *n* of our measurements. Then, we calculated the median of the variances of the replicates for each *n* and plotted this median against sample size, revealing a breakpoint in this relationship. This breakpoint indicates the minimum number of measurements (*n*) necessary to explain the same amount of variance as using the whole population of measurements. Breakpoints, trends and their statistical significance were estimated using a sequential Mann-Kendall analysis. For these analyses, stem emissions from UpperStem and LowerStem were pooled together for each gas.

Additionally, for each GHG flux we calculated linear correlations between tree locations in order to provide insights on potential sources of stem-gas emissions and/or potential transport paths. WCA were performed using MATLAB R2010b (The MathWorks Inc.) and all the other analyses were carried out using R 3.4.3 (R Foundation for Statistical Computing, Vienna, Austria). GLSs models were performed using the R nlme package^79^, model selection and comparison was done with the R MuMIn package^80^, and breakpoint analysis of flux variance trends with sample size was carried out with the R greenbrown package^81^.

## Supplementary information

Supplementary Material
