# Supplementary material for: Automated measurements of greenhouse gases fluxes from tree stems and soils: magnitudes, patterns and drivers
Source: Sci Rep. 2019 Mar 8;9:4005. doi: 10.1038/s41598-019-39663-8 (PMC6408546; doi:10.1038/s41598-019-39663-8)
Supplement: Supplementary file 1 — Supplementary Material [file 41598_2019_39663_MOESM1_ESM.pdf]

Automated measurements of greenhouse gases fluxes from tree stems and soils: magnitudes, patterns and drivers

Supplementary Information

Authors:

Josep Barba[1], Rafael Poyatos[2,3] and Rodrigo Vargas[1*]

[1] Department of Plant and Soil Science. University of Delaware. Newark, Delaware, 19717 USA

[2] CREAF. Cerdanyola del Vallès 08193 (Barcelona), Catalonia, Spain

[3] Laboratory of Plant Ecology. Faculty of Bioscience Engineering. Ghent University. Ghent, 9000 Belgium

* Corresponding author:

Rodrigo Vargas

Email: rvargas@udel.edu

Phone: 302-831-1386, Fax: 302-831-0605

SFigure 1. Seasonal course of CO_2, CH_4 and N_2O fluxes (panels a, b and c, respectively) associated to UpperStem (black), LowerStem (blue) and Soil (red) chambers. Points are hourly means taken from Day of the Year 102 to 202 (April 12th 2017 - July 21st 2017) and lines depict smoothed temporal patterns for CO_2, CH_4 and N_2O fluxes.

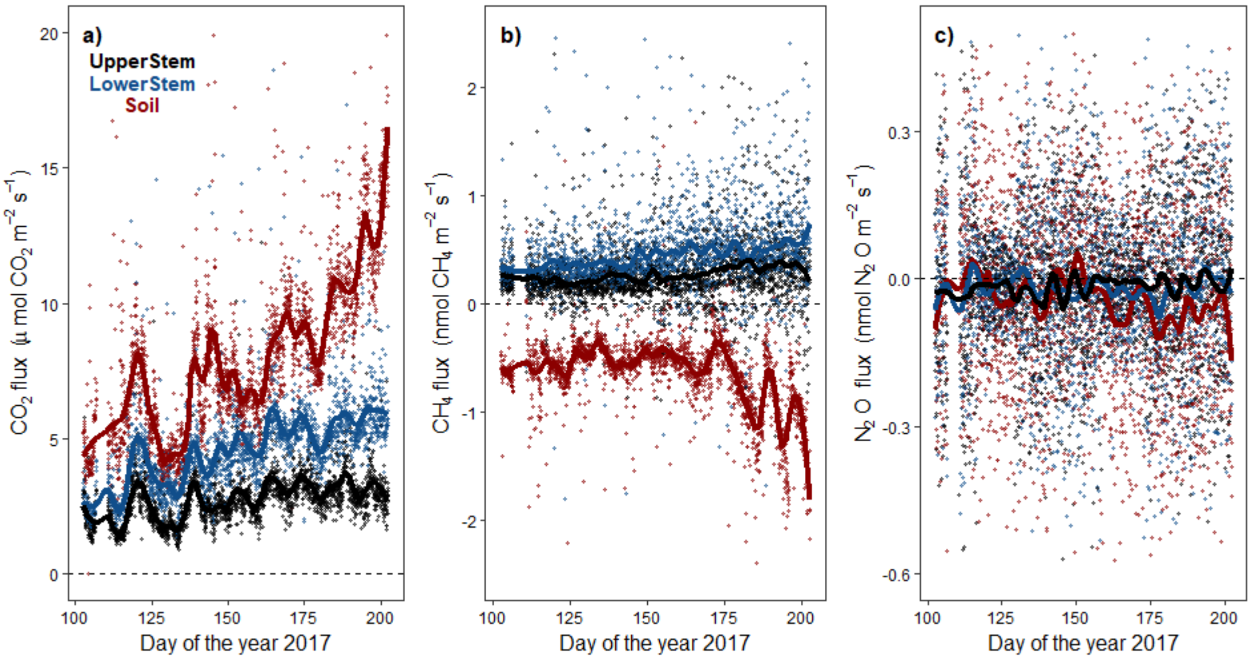

STable 1. Summary of the models differing less than 2 AICc units from the selected model for each greenhouse gas (CO_2, CH_4 and N_2O) and each position (UpperStem, LowerStem and Soil). All variables were scaled to improve the performance and interpretability of the models.

MODEL	Variables	Estimate	SE	t-value	p-value
Soil CO_2	(Intercept)	0.162	0.254	0.639	0.525
**selected model**	Temperature	1.011	0.116	8.684	<0.001
adjR^2 = 0.99	SWC	0.174	0.095	1.825	0.072
AICc = 47.89	SF	0.081	0.036	2.255	0.027
	Temp*SWC	-0.240	0.072	-3.315	0.001
Soil CO_2	(Intercept)	0.282	0.380	0.742	0.460
Model 2	Temperature	1.011	0.122	8.317	< 0.001
adjR^2 = 0.99	SWC	0.249	0.100	2.494	0.015
AICc = 51.82	SF	0.091	0.036	2.568	0.012
	Temp*SWC	-0.250	0.073	-3.441	< 0.001
	Temp*SF	-0.062	0.034	-1.800	0.076
Soil CO_2	(Intercept)	0.190	0.157	1.207	0.231
Model 3	Temperature	0.928	0.103	9.052	< 0.001
adjR^2 = 0.98	SWC	0.071	0.073	0.975	0.333
AICc = 45.43	Temp*SWC	-0.160	0.055	-2.890	0.005
LowerStem CO_2	(Intercept)	0.106	0.142	0.747	0.457
**selected model**	Temperature	0.517	0.095	5.446	<0.001
adjR^2 = 0.92	SWC	0.192	0.104	1.841	0.069
AICc = 98..44	SF	0.159	0.049	3.266	0.002
	Temp*SWC	0.206	0.079	2.606	0.011
	Temp*SF	-0.254	0.044	-5.723	<0.001
LoweStem CO_2	(Intercept)	0.139	0.123	1.139	0.258
Model 2	Temperature	0.501	0.089	5.609	< 0.001
adjR^2 = 0.92	SWC	0.211	0.108	1.949	0.055
AICc = 103.53	SF	0.189	0.059	3.194	0.002
	Temp*SWC	0.226	0.086	2.642	0.010
	Temp*SF	-0.255	0.045	-5.611	< 0.001
	SWC*SF	-0.055	0.072	-0.757	0.451
UpperStem CO_2	(Intercept)	-0.113	0.125	-0.909	0.366
**selected model**	Temperature	0.634	0.084	7.578	< 0.001
adjR^2 = 0.93	SWC	0.566	0.106	5.351	< 0.001

AICc = 93.90	SF	-0.001	0.058	-0.002	0.998
	Temp*SF	-0.200	0.045	-4.456	< 0.001
	SWC*SF	0.318	0.067	4.772	< 0.001
Soil CH_4	(Intercept)	-0.142	0.090	-1.580	0.118
**selected model**	Temperature	-0.666	0.085	-7.822	<0.001
adjR² = 0.90	SWC	0.761	0.076	9.966	<0.001
AICc = 108.33	Temp*SWC	0.427	0.065	6.540	<0.001
LowerStem CH_4	(Intercept)	0.002	0.096	0.021	0.983
**selected model**	Temperature	0.447	0.097	4.613	<0.001
adjR² = 0.34	SWC	0.279	0.096	2.903	0.005
AICc = 236.34					
LowerStem CH_4	(Intercept)	0.000	0.092	-0.002	0.999
Model 2	Temperature	0.328	0.103	3.177	0.002
adjR² = 0.31	SF	0.298	0.103	2.907	0.005
AICc = 239.96					
LowerStem CH_4	(Intercept)	0.000	0.091	0.002	0.998
Model 3	Temperature	0.364	0.103	3.536	0.001
adjR² = 0.35	SWC	0.252	0.114	2.217	0.029
AICc = 239.65	SF	0.132	0.124	1.064	0.290
UpperStem CH_4	(Intercept)	0.001	0.088	0.010	0.992
**selected model**	Temperature	0.451	0.098	4.488	< 0.001
adjR² = 0.39	SF	0.254	0.097	2.620	0.01
AICc = 227.99					
UpperStem CH_4	(Intercept)	-0.091	0.092	-0.991	0.325
Model 2	Temperature	0.465	0.098	4.731	< 0.001
adjR² = 0.40	SF	0.289	0.095	3.028	0.003
AICc = 230.70	Temp*SF	0.144	0.098	1.470	0.145
UpperStem CH_4	(Intercept)	-0.081	0.097	-0.844	0.401
Model 3	Temperature	0.459	0.100	4.488	< 0.001
adjR² = 0.40	SWC	-0.045	0.128	-0.351	0.726
AICc = 234.85	SF	0.313	0.119	2.638	0.010
	Temp*SF	0.140	0.099	1.415	0.161
UpperStem CH_4	(Intercept)	-0.019	0.088	-0.211	0.833
Model 4	Temperature	0.440	0.101	4.339	< 0.001
adjR² = 0.39	SWC	-0.060	0.129	-0.464	0.644

AICc = 232.03	SF	0.307	0.120	2.559	0.012
Soil N_2O	(Intercept)	-0.006	0.131	-0.045	0.964
**selected model**	Temperature	-0.382	0.129	-2.960	0.004
$adjR^2 = 0.22$					
AICc = 243.92					
Soil N_2O	(Intercept)	-0.001	0.132	-0.009	0.993
Model 2	Temperature	-0.432	0.138	-3.119	0.003
$adjR^2 = 0.23$	SWC	0.138	0.131	1.047	0.298
AICc = 247.04					
Soil N_2O	(Intercept)	-0.004	0.128	-0.033	0.974
Model 3	Temperature	-0.435	0.148	-2.946	0.004
$adjR^2 = 0.22$	SF	0.089	0.128	0.697	0.488
AICc = 247.74					
LowerStem N_2O					
**selected model**					
p-value = n.s.					
AICc = 259.57					
LowerStem N_2O	(Intercept)	-0.003	0.117	-0.025	0.980
Model 2	SF	-0.179	0.112	-1.599	0.113
$adjR^2 = 0.04$					
AICc = 261.61					
UpperStem N_2O	(Intercept)	0.003	0.114	0.026	0.980
**selected model**	Temperature	0.240	0.126	1.899	0.061
$adjR^2 = 0.13$	SWC	0.260	0.136	1.923	0.058
AICc = 261.28	SF	-0.404	0.143	-2.816	0.006
UpperStem N_2O	(Intercept)	0.045	0.153	0.295	0.769
Model 2	Temperature	0.166	0.141	1.181	0.241
$adjR^2 = 0.14$	SWC	0.262	0.201	1.303	0.196
AICc = 263.67	SF	-0.403	0.153	-2.631	0.010
	SWC*SF	-0.194	0.138	-1.413	0.161
UpperStem N_2O	(Intercept)	0.085	0.159	0.537	0.593
Model 3	SWC	0.218	0.205	1.062	0.291
$adjR^2 = 0.13$	SF	-0.335	0.142	-2.364	0.020
AICc = 260.85	SWC*SF	-0.237	0.140	-1.694	0.094

UpperStem N_2O	(Intercept)	-0.030	0.162	-0.199	0.843
Model 4	Temperature	0.196	0.141	1.394	0.167
adjR^2 = 0.16	SWC	0.282	0.200	1.413	0.161
AICc = 266.35	SF	-0.398	0.152	-2.615	0.011
	Temp*SF	0.160	0.125	1.281	0.204
	SWC*SF	-0.185	0.136	-1.354	0.179
UpperStem N_2O	(Intercept)	-0.153	0.130	-1.175	0.243
Model 5	Temperature	0.232	0.134	1.740	0.085
adjR^2 = 0.15	SWC	0.427	0.164	2.594	0.011
AICc = 263.79	SF	-0.457	0.144	-3.163	0.002
	Temp*SF	0.168	0.124	1.356	0.179
UpperStem N_2O	(Intercept)	-0.147	0.130	-1.128	0.262
Model 6	Temperature	0.246	0.135	1.819	0.073
adjR^2 = 0.15	SWC	0.419	0.165	2.535	0.013
AICc = 267.02	SF	-0.458	0.145	-3.159	0.002
	Temp*SWC	0.192	0.130	1.472	0.145
	Temp*SF	-0.108	0.180	-0.599	0.551
